# Research progress and strategies for the biosynthesis of keratinocyte growth factor-2 (KGF-2) in *Arabidopsis thaliana*

**DOI:** 10.7717/peerj.19440

**Published:** 2025-05-27

**Authors:** Chengyang Song, Guangdong Yu, Shuang Gao, Wengang Zhao, Yunpeng Wang, Nuo Xu

**Affiliations:** 1 Jilin Academy of Agricultural Sciences (Northeast Agricultural Research Center of China), Institute of Agriclutural Biotechnology, Changchun, Jilin Province, China; 2 Wenzhou University, College of Life and Environmental Science, Wenzhou, Zhejiang Province, China; 3 Wenzhou Medical University, School of Pharmaceutical Sciences, Wenzhou, Zhejiang Province, China; 4 Zhejiang Tianqu Beiben Instrument Technology Co., Ltd., Wenzhou, Zhejiang Province, China

**Keywords:** Molecular farming, KGF-2, Skin care, Hair growth, Transdermal peptide, Plant, Arabidopsis thaliana

## Abstract

This review examines the current research progress of molecular agriculture for cytokine expression, the research status of keratinocyte growth factor-2 (KGF-2) for skin care, hair growth, and the research status of plants for hair growth. KGF-2 application and use are inhibited by factors, such as the cost of production and efficacy due to low transdermal penetration potential. However, cell penetrating peptides (CPPs) are powerful transporter tools that assist the transmembrane delivery of attached biomacromolecules. Biomacromolecules such as KGF-2 have poor transdermal ability, which can be enhanced by combining with CPP such as TD1 to construct fusion proteins. *Arabidopsis thaliana* has many advantages, such as short growth cycle, high transformation efficiency, large biomass and hair growth promotion substances. Therefore, we also discuss the feasibility and expression strategies of using *A. thaliana* plant system to express fusion protein TDP1-KGF-2, which provides new idea for enhancing the transdermal ability of KGF-2 and providing new idea for plant system expression of fusion protein. This offers a promising new direction for the development of natural hair care and hair growth materials, as well as innovative possibilities for the application of molecular agriculture.

## Introduction

Molecular agriculture is widely used in the research and production of antibodies, vaccines, *etc*. The expression of keratinocyte growth factor-2 (KGF-2) and other cytokines in plant bioreactors has many advantages such as higher safety. KGF-2 can regulate cell proliferation, differentiation and migration (Vega-Hernández et al., 2011), and play a key role in wound healing, scar repair and hair growth (Zhou et al., 2022; Ergen et al., 2022; Zhang et al., 2018). However, KGF-2 has a high molecular weight and poor transdermal ability. However, transdermal peptides can assist biological macromolecules such as insulin and epidermal growth factor (EGF) to penetrate the skin barrier and significantly improve the transdermal ability (Chen et al., 2006; Quan Ruan et al., 2013; Kerkis et al., 2006). Therefore, it is feasible to combine the transdermal peptide with KGF-2 to construct a fusion protein to enhance the transdermal ability of KGF-2. The expression of fusion proteins in plant expression systems has many advantages, such as post-translational modification. At present, there are relatively few studies on the use of plant bioreactors to express fusion proteins. The extracts of a variety of plants have the effect of promoting hair growth and contain effective ingredients for promoting hair growth, which have been reviewed and summarized (Park & Lee, 2021). *Arabidopsis thaliana* is a model plant with advantages such as fast growth rate and high transformation efficiency, and is an ideal receptor for exploring the expression of fusion proteins (Zheng et al., 2021; Mattioli et al., 2019). At the same time, *A. thaliana* contains effective ingredients such as shikimic acid to promote hair growth, and exploring their synergistic effect with fusion protein in promoting hair growth can provide a new direction for the development of related products (Park & Lee, 2021).

In this review, we summarize the related research on the expression of cytokines in molecular agriculture, the biological function and research status of KGF-2, and explore the expression strategy of fusion protein TDP1-KGF-2 in *A. thaliana*. The expression of fusion protein in *A. thaliana* can explore the synergistic effect of fusion protein and active components in plants, and explore a new direction for the development of molecular agriculture. At the same time, the expression of fusion protein through plants can meet the society’s pursuit of pure natural for drugs and cosmetics and so on.

## Review Methodology

The data presented herein were acquired following a rigorous search from several well-established academic search engines and specific journal databases. These included PubMed, Google Scholar, Web of Science, and Baidu Academic Search. There are differences in the availability of relevant papers among different databases. PubMed and Google Scholar were the main search tools, and their comprehensiveness was confirmed by preliminary search, and most relevant studies could be obtained. To verify the coverage, supplementary searches were also conducted in Web of Science and Baidu Academic Search. These complementary searches further confirmed that PubMed and Google Scholar provided the most extensive and relevant results. To optimize and find relevant literature, we used Boolean search method and keywords related to the following review topics: molecular agriculture, cytokine, plant bioreactor, KGF-2, biological function, hair growth, transdermal peptide, plant extract, *A. thaliana* extract, transgene, fusion protein. The Boolean operator (AND, OR) is used to optimize the search and ensure the comprehensive coverage of the search results. Medical subject headings (MeSH) were used in PubMed to improve the precision of the search. Study inclusion criteria were reviews and original research papers published in English, *etc*. Titles and abstracts of identified reports were screened for relevance. Subsequently, the full text of potentially relevant reports was reviewed in detail to assess their suitability for the topic and content of this review, thereby ensuring the quality and accuracy of interpretations of the findings and conclusions described herein.

## Molecular Farming in Cytokine Expression

Molecular farming, also known as plant bioreactors, refers to the use of gene editing technology to express metabolites or proteins in plants for research or industrial use (Kowalczyk et al., 2022). Molecular farming is an interdisciplinary approach to biosynthesis used in medicine, agronomy, and engineering. The rapid progress in molecular farming over the past decades has led to its wide use in the development and research of healthcare and beauty products (Shanmugaraj, I. Bulaon & Phoolcharoen, 2020). Notably, plant bioreactors have been used to produce antibodies and vaccines. With advantages such as high safety and stable protein expression, molecular farming has been widely accepted as a successful method for synthesizing proteins (dos Santos et al., 2024). At present, the established plant production platforms mainly include root hair, cell suspension culture, and whole plant platform (Huang & McDonald, 2012). Proteins in these platforms are expressed following the transformation of the plant or plant cells *via* stable nuclear transformation, transient transformation, or chloroplast transformation (Krenek et al., 2015; Liu et al., 2023).

The expression of cytokines using plant bioreactors has been heavily investigated, to reduce the cost of commercially available recombinant cytokines (Yu et al., 2024). Various proteins have been expressed in plants. For example, human IL-13 and GM-CSF have been successfully expressed in tobacco plants or cell cultures (Kim, Kwon & Moon Sik, 2004; Wang et al., 2008). Additionally, growth factors, such as aFGF, bFGF, and epidermal growth factor (EGF) have also been expressed in plants (Yu et al., 2024). Ding et al. (2006) successfully expressed the human basic fibroblast growth factor (bFGF) in soybean seeds using the G1 promoter. The synthesized bFGF returned its biological activity as it promoted mitosis in Balb/c 3T3 cells. Qiang et al. (2020) expressed EGF in *A. thaliana* using the oleosin fusion technology, thus managing to express recombinant EGF in oil bodies. These data suggest that plants are feasible bioreactors for the synthesis of various cytokines and growth factors.

There are several advantages of plant bioreactors. First, genes introduced into plants are stably inherited and less likely to be lost due to plasmid loss, a phenomenon commonly observed in prokaryotic systems (Yu et al., 2024). Second, proteins expressed in plants can be continuously obtained, as plants can be cultivated using conventional methods, such as open-air cultivation, greenhouse, and vertical farms. This increases the scalability of production and reduces cost (Huebbers & Buyel, 2021). Third, plants have the post-translation modification machinery, which allows the modification of expressed proteins and ensures the retention of the structure and function of the protein (Huang & McDonald, 2012). Fourth, compared with prokaryotic, yeast and animal cell expression systems, the requirements of plants and plant cell growth are simpler, and there is no heat source, endotoxin or other sensitive substances, and there is no risk of contamination by pathogens. Therefore, the plant expression system reduces some potential sensitization and pathogenic risks of synthesizing proteins, thereby making plant-synthesized proteins safer (Yu et al., 2024; Moustafa, Makhzoum & Trémouillaux-Guiller, 2016). Fifth, the extraction, separation, and purification of proteins expressed in plants does not require complex processes. Taken together, plants are superior bioreactors compared to commonly used expression systems, such as the prokaryotic expression system. The scalability of production together with the simplicity of the extraction and purification further makes plants a better commercial alternative for the production of high-value proteins. Furthermore, as people are increasingly looking for natural sources of raw materials used in cosmetics and health products, plant bioreactors are likely to satisfy this desire.

The application of plant bioreactors also has some problems and challenges that need to be addressed. Some plant systems suffer from low production of exogenous proteins, which makes the commercial application of plant bioreactors difficult. Compared with mammalian cells, there are differences in post-translational modification mechanisms and concurrent endoplasmic reticulum stress problems. There are also difficulties in the extraction and purification of exogenous proteins from plants. Plant expression systems also face legal regulation and safety issues, such as the need to ensure that the environment is free from the invasion of transgenic plants caused by reproductive structures such as seeds (Quan Ruan et al., 2013). These challenges are being overcome. Precise gene-editing technologies such as CRISPR-Cas (CRISPR-associated system) can be used to address issues such as gene flow and endoplasmic reticulum stress (Burnett & Burnett, 2020). Improving the bioactivity of exogenous proteins by optimizing glycosylation modification (Obembe et al., 2011; Schillberg & Spiegel, 2022). It can further reduce the cost and improve the efficiency of protein extraction by optimizing the planting conditions and protein extraction process, and further enhance the market feasibility. Various methods such as optimizing codon bias, optimizing expression vectors, selecting appropriate regulatory elements, and applying gene editing technology are used to improve the consistency of exogenous protein production and reduce immunogenicity (Yu et al., 2024; Moustafa, Makhzoum & Trémouillaux-Guiller, 2016; Obembe et al., 2011; Schillberg & Spiegel, 2022).

Taken together, plant expression systems are superior bioreactors. The plant expression system is highly safe and does not require complex fermentation equipment and other conditions. The stable expression and accumulation of foreign proteins can be achieved by optimizing planting conditions and gene expression regulation strategies. The plant expression system has advantages over other expression systems for industrial production. A number of advantages make plants a better commercial choice for the production of high-value proteins. Furthermore, as people are increasingly looking for natural sources of raw materials used in cosmetics and health products, plant bioreactors are likely to satisfy this desire.

## Functions of KGF-2

Keratinocyte growth factor-2 (KGF-2), also known as fibroblast growth factor-10 (FGF-10), is a member of the fibroblast growth factor family. KGF-2 is a heparin-binding protein composed of 208 amino acids with a molecular weight of about 24 kDa, which has strong regenerative properties; is a multifunctional growth factor produced by mesenchymal cells and acts on epithelial cells in a paracrine manner to mainly promote proliferation, migration, and differentiation (Bi et al., 2014; Wang et al., 2016; Vega-Hernández et al., 2011). Given this wide range of functions, KGF-2 can play a key role in fetal limb development, and lung development as well as injury repair, wound healing, scar repair, hair growth, *etc.*

### KGF-2 in skin wound healing

Wound healing is a complex physiological process, which can be divided into the inflammatory phase, proliferative phase, and remodeling phase (Xiaojie et al., 2022). Each of these phases requires intricate coordination to avoid exorbitant activation, which leads to faulty healing. During the inflammatory phase, KGF-2 regulates the inflammatory response and inhibits the overexpression of inflammatory factors (Braun et al., 2004). During the proliferative phase, KGF-2, *via* its FGF receptor 2 IIIb (FGFR2 IIIb) signaling, has been shown to play a vital role in skin wound healing where it not only promotes the proliferation of keratinocytes but also regulates the phases of tissue healing (Chen et al., 2009; He et al., 2024; Fang, Bai & Wang, 2010). During the remodeling phase, KGF-2 inhibits STAP-2 expression and signal transducer and activator of transcription 3 activation, resulting in a significant reduction in collagen I and III levels, thereby reducing scar formation while facilitating wound healing (Zhou et al., 2022). Smith et al. (2000) found that KGF-2 significantly increased the interstitial closure rate of human meshed skin graft on a “nude” rat model, significantly promoting wound healing. Observations by Gao et al. (2021) also showed that pretreatment of the epidermis with KGF-2 mitigates the degree of skin photodamage by inhibiting cell apoptosis, reducing oxidative stress and regulating AhR/Nrf2 signaling pathway, which in turn prevents DNA damage and mitochondrial dysfunction. Taken together, these findings indicate that KGF-2 facilitates wound healing while reducing scar formation making it a potential anti-scarring therapeutic.

### KGF-2 in eye injury repair

Corneal injury is a lesion caused by either trauma or exposure to external stimuli, that disrupts the corneal epithelium leading to symptoms such as pain, burning, and tearing of the eye. The process of corneal injury repair can be divided into the lag or latent, migration, proliferation and differentiation, and the reattachment and matrix remodeling phase. In the initial stages of the process, inflammatory cells migrate to the wound site causing inflammation, redness, swelling, and blurred vision. Naive epithelial cells then migrate to the wound site, proliferate and differentiate to form a new epithelial layer that seals the wound site, gradually leading to healing. Complications such as microbial infection or ulcer formation may affect the outcome of corneal healing as they can lead to loss of corneal transparency. The role of KGF-2 in corneal injury repair has been explored using various models. KGF-2 promotes the proliferation and differentiation of epithelial cells, promotes epidermal regeneration, and accelerates corneal wound healing. Yang, Fu & Li (2002) showed that in chronic wounds of adults, KGF-2 accelerates wound closure faster than EGF as its promotion of keratinocyte proliferation is significantly higher. Using the rat corneal alkali burn model, Cai et al. (2019) compared the efficacy of bFGF and KGF-2 in preventing excessive wound healing and scar formation. Observations from this study showed that KGF-2 is a significantly better facilitator of epidermal regeneration, migration, and scar formation reduction, thus suggesting it can be used to treat corneal injury. Similarly, Ergen et al. (2022) showed that KGF-2 effectively improves early reepithelialization, opacification, and neovascularization while reducing inflammation in corneal alkali burn. Subsequent related studies also showed that KGF-2 stimulates limbal epithelial stem cells to migrate to the central cornea, thus, accelerating the healing of alkali burn cornea (Liu et al., 2005). KGF-2 activates and regulates p38, ERK1/2, Nrf2 and P13K signaling pathways to promote the repair of corneal injury (Xiaojie et al., 2022). KGF-2 could induce cell proliferation and migration by activating ERK1/2 and p38 signaling pathways (Cai et al., 2019). Liu et al. (2022) have shown that KGF-2 can prevent cataracts by regulating Nrf2/hydroxide ion and PI3K/Akt pathways, eliminating oxidative stress and apoptosis induced by hydrogen peroxide (hydrogen peroxide monovalent anion) in human lens epithelial cells (HLECs) and rat lenses.

### KGF-2 in promoting hair regeneration

Hair is not just a defining characteristic of mammals but is also regarded as a natural ornament, hence, it plays an important role in personal appearance and perception. Hair also confers other protective functions, such as protecting the scalp from ultraviolet rays, low temperature, and external factors. Hair growth is a complex cyclic and periodically controlled process characterized by rapid growth (anagen), regression (catagen), and resting periods (telogen) (Liu et al., 2016). This process is regulated by many factors, such as genetics, nutrition, environmental factors, hormones, and individual health status. Hair follicles are composed of epidermal and dermal (mesenchymal) compartments, whose interaction is essential for the maintenance of the morphology and growth of hair follicles. At the bottom of hair follicles are dermal cells surrounded by the hair matrix (HM), which induce and regulate hair growth by modulating signals across the entire hair follicle epithelium. These dermal cells are also known to release growth factors, such as insulin-like growth factor-1 (IGF-1) and vascular endothelial growth factor (VEGF), which also influences hair growth (Itami, Kurata & Takayasu, 1995; Lachgar et al., 1999). Other growth factors have also been demonstrated to influence hair growth. For example, KGF and hepatocyte growth factor (HGF) have stimulatory effects on hair follicle growth while EGF and transforming growth factorβ-(TGF-β) have inhibitory effects on hair follicle growth (Roh et al., 2002; Lichtenberger, Mastrogiannaki & Watt, 2016). Among the members of the FGF family, FGF-10 is known to significantly promote hair growth (Xiaojie et al., 2022). In wound healing, FGF-10 promotes hair follicle cell proliferation and migration and induces anagen. Similar observations have been made in nude mice where FGF-10 binds FGFR2IIIb to increase and grow hair follicles in nude mice (Petiot et al., 2003). The regulation of hair follicle morphological development and cycles involves a variety of different molecular signaling pathways, mainly including Wnt/β-catenin signaling pathway, sonic hedgehog (SHH) signaling pathway, Notch signaling pathway and so on Wang et al. (2022). Wingless/Integrated (WNT) signaling protein is mainly expressed during hair follicle anagen, decreased during catagen, and inactivated during telogen (Lin, Zhu & He, 2022). SHH is a small secreted glycoprotein, that is involved in the regulation of follicle cycling and promotes the transition of hair follicles from telogen to anagen (Gao et al., 2019). KGF-2 can regulate hair follicle development by activating Wnt/β-catenin and SHH signaling pathways. A recent study by Zhang et al. (2018) showed that FGF-10 and frizzled-related protein 1(sFRP1) compete to regulate the development of hair follicles. In the study, FGF-10 was shown to promote hair growth and regeneration by increasing the nuclear levels of β-catenin (Zhang et al., 2018). Jang (2005) found that the application of recombinant human KGF-2 (rhKGF-2) can significantly stimulate the proliferation of human hair follicle cells (26–35 perent). Among all commercial FGFs approved by the State Food and Drug Administration (SFDA) for wound healing, KGF-2 is the best candidate for stimulating hair growth of hair follicles at the wound site (Xiaojie et al., 2022). KGF-2 has the highest rate of hair growth stimulation compared with FGF-1 and FGF-2 (Lin et al., 2015). While FGF-1 and FGF-2 are capable of inducing hair growth, the induction of hair growth is delayed, ununiform, and only occurs at proximal regions. Mice treated with KGF-2 show a more uniform hair growth with longer hair length (Lin et al., 2015). At the same time, related studies have shown that KGF-2 can induce hair growth by up-regulating the expression of Wnt/β-catenin and sonic hedgehog (SHH), and this up-regulation begins to decrease at 3 and 4 weeks, leading to the end of hair follicle growth phase. Continuous stimulation keeps the hair follicles in the growth phase, which may lead to the development of hair follicles in the direction of tumors (Lin et al., 2015). The poor transdermal absorption properties of KGF-2 impede its promotion of hair regeneration. To overcome this limitation, recombinant KGF-2 have been fused to other molecules with better transdermal properties. Li et al. (2017) fused rhFGF-10 to an oil body gene, oleosin-rhFGF-10 protein, and this enhanced the protein’s stability and effect. Liu et al. (2016) used the dimer oil-globulin fusion method to express KGF-2 and verified its biological activity, and found that KGF-2 could be expressed by this method, and the asymmetric O-O:: KGF-2 construct had the highest expression and biological activity. Compared with recombinant FGF-10 expressed in prokaryotic systems, recombinant FGF-10 (oleosin-rhFGF-10) expressed in plants (such as safflower) is more effective in promoting skin wound healing and hair growth in mice (Li et al., 2017).

## Strategies for KGF-2 biosynthesis

### KGF-2 fusion expression strategy

Although KGF-2 plays an important role in hair growth, wound healing, and embryonic development, its short half-life, poor thermal stability, and low transdermal properties limit its potential clinical application, especially in tropical regions. At the same time, the production of KGF-2 faces the problems of low expression, high cost, insufficient post-translational modification, and complex technology. To overcome these limitations, various methods of expressing and modifying KGF-2 have been employed. For example, hKGF-2, containing 208 nucleic acids, has been subcloned into pET-30a(+) and successfully expressed in a prokaryotic system, the yield was approximately 2.2 g/L (Kim et al., 2023). To improve its solubility, FGF-10 can be fused with the Halo-tag and expressed (Sun et al., 2015). Full-length hFGF-10 has also been subcloned into pPICZα and expressed in Pichia pastoris GS115, the yield was approximately 1.0 g/L (Giddings et al., 2000). The acquired rhFGF-10 was shown to promote the proliferation of NIH-3T3 (Giddings et al., 2000). Although the rhFGF-10 showed similar biological functions to those of natural FGF-10, modified FGF-10 is still prone to denaturation. Thus, needing further modification to increase stability. The fusion of oil body-protein or dimeric oil-globulin fusion has been shown to reduce protein denaturation. Furthermore, the fusion of a protein to an oil body increases its transdermal properties. Some researchers used oil body-protein system and dimeric oil-globulin fusion method to express KGF-2, and used plant bioreactors to obtain bioactive KGF-2 (Liu et al., 2016; Li et al., 2017). Construct a plant expression vector containing the KGF-2 gene, combined with the oleosingene or other genes, and then transfer the vector into Agrobacterium tumefaciens. Seeds were screened after transforming plants using the floral dip technique. T3 seeds were screened to obtain the single copy homozygous generation. These methods can enhance the stability of the expressed protein, which is conducive to long-term expression and application; moreover, the transdermal ability of KGF-2 combined with oil body is also enhanced compared with that of KGF-2 expressed in prokaryotic system (Liu et al., 2016; Li et al., 2017). KGF-2 has been expressed in prokaryotic expression systems, yeast expression systems and plant expression systems. Different expression systems have different advantages and disadvantages for expressing foreign proteins. Table 1 presents the potential advantages and disadvantages of KGF-2 expression by different expression systems (dos Santos et al., 2024; Liu et al., 2016; Li et al., 2017; Kim et al., 2023; Sun et al., 2015; Giddings et al., 2000).

**Table 1:** Advantages and disadvantages of KGF-2 expression by different expression systems.

Expression system	Advantages	Disadvantages
Bacteria	Easy to manipulate	Poor folding capacity
	Low cost	Endotoxin accumulation
	High expression	Lack of post-translational modifications
	Short turnaround time	
	Ease of scale up	
Yeast	Easy to manipulate	The thick and hard cell walls
	High expression	Medium turnaround time
	Rapid growth and scalable	Low endotoxin content
	Simple culture conditions	Hyperglycosylation of proteins
	With post-translational modifications	Limited glycosylation capacity
Plant	Low cost	Low expression
	Optimized growth conditions	Long turnaround time
	Free from pathogen contaminants	Limited glycosylation capacity
	Free from bacterial toxin contaminants	
	With post-translational modifications	
	The oil body fusion strategy was used	

Taken together, the use of a fusion strategy to express KGF-2 can increase its half-life and solubility without affecting its biological activity and this may lead to increased clinical efficacy and application.

Skin is the largest organ of the human body, which constructs the first barrier of the body’s defense (Zhang et al., 2021). Due to the existence of the skin barrier, most biological macromolecules are unable to effectively penetrate the skin barrier. This not only hinders the penetration of harmful substances but also of drugs making them less effective (Hänel et al., 2013). Various transdermal drug delivery systems have been used to increase the penetration of drugs across the skin barrier, and the right delivery system or method can make active ingredients such as drugs work better (Zhang et al., 2021). Transdermal delivery methods can be divided into physical, chemical, or biological methods. Physical methods include electroporation, microneedle introduction, ultrasonic treatment, and iontophoresis. While these methods can promote quick penetration of the drug to the dermis, they cause discomfort to the user (Bok et al., 2023; Dermol-Černe, Pirc & Miklavčič, 2020; Hong et al., 2018). Chemical methods are based on the construction of transfer bodies, microcarriers, and capsules that are easy to penetrate the skin to carry drugs across the skin barrier. While this does not cause discomfort to the user, it often results in changes in the physical and chemical properties of the active ingredient, and certain chemicals have potential known and unknown hazards to the skin (Kalvodová & Zbytovská, 2022; Akram et al., 2022; Damiani & Puglia, 2019). The biological methods include the coupling or fusing of the molecule of interest with screened suitable biomolecule(s) known to have higher transdermal properties (penetrating peptides). This results in the increase of the penetrating potential of the molecule of interest without changing its structure and function (Chablani & Singh, 2022). Transdermal peptides have been widely used in peptide-protein fusion research for pharmacologically significant proteins. Compared to other transdermal delivery strategies, the biological methods have a higher biological safety profile.

Cell-penetrating peptides (CPPs) are effective transdermal delivery systems and powerful transporter tools capable of penetrating the cell membrane and delivering attached biomacromolecules into cells or the cell-microenvironment (Pujals et al., 2006). Cell-penetrating peptides are short peptides with unique cell membrane penetration ability. Currently, hundreds of penetrating peptides have been discovered, of which, several have been used to overcome the skin barrier (Kerkis et al., 2006). Penetrating peptides can be used to mediate the delivery of bimolecular substances such as proteins, peptides, and drug-loaded nanoparticles, and they do not require receptors *in vivo* and *in vitro*, and do not cause obvious membrane damage, which also makes penetrating peptides attract great attention in drug research (Nasrollahi et al., 2012). The cytotoxicity of penetrating peptides is also quite low and they will eventually degrade to amino acids; a large number of penetrating peptides with different sequences have been identified to have different transdermal mechanisms and can transport different substances (Reissmann, 2014). The cellular uptake mechanisms of macromolecules delivered by CPP can be divided into direct penetration and endocytic routes, which are affected by many factors such as experimental factors, cell types, and so on (Reissmann (2014) and Sun et al. (2023)). Among these peptides, Translocating peptide 1 (TP1) and Transdermal peptide 1 (TD1) have been heavily investigated and their mechanisms are fairly understood. TD1, a transdermal peptide identified by high-throughput screening, is a peptide chaperone consisting of the ACSSSPHKHCG sequence that facilitates the transdermal delivery of multiple proteins using topical combination delivery. TD1 can help biomacromolecules penetrate the skin through specific binding to the Na, K-ATPase beta subunit (ATP1B1), energy-dependent mechanism, and skin appendages pathway (Ruan et al., 2016). TD1 has been used to enhance the transdermal properties of growth factors such as epidermal growth factor. Ruan et al. fused TD1 with human epidermal growth factor (hEGF) and expressed it in yeast system. Compared with hEGF, the transdermal performance of the fusion protein was significantly improved, and the transdermal mechanism of TD1 was explored at the animal level, thus laying a foundation for the application of TD1 as a transdermal delivery vector (Ruan et al., 2014). Additionally, Jin et al. (2014) fused EGF with double TD1 motifs and showed that both the TD1-hEGF-TD1 and TD1-TD1-hEGF fusion pattern result in further enhancement of the transdermal properties of EGF by 5 times compared to hEGF and 1 time compared to TD1-hEGF. This laid the foundation for the use of TD1 to improve the transdermal ability of proteins and other active substances. The structure and transdermal mechanism of TP1 have been explored by Muñoz-Gacitúa, Guzman & Weiss-López (2022), using nuclear magnetic and isotope labeling methods, and the peptide has been evaluated for its delivery of antitumor drugs. TP1 has been used to deliver active substances such as cabazitaxel in the treatment of prostate cancer and breast cancer (Park et al., 2021). The promising effects of CPPs in transdermal delivery suggest that they are poised to become a preferred bio-transdermal method for hair growth products.

In skincare and haircare research and products, transdermal peptides are used as biological delivery systems and combined with target proteins or peptides forming fusion proteins or peptides. As KGF-2 is a good candidate drug for hair growth, fusing it with transdermal peptide may help in overcoming its low transdermal ability and its thermal instability. This may lead to more clinical applications of KGF-2 even beyond hair growth and skincare.

### Plants extracts for hair growth and care

Hair loss, or other problems associated with hair growth, have multifactorial effects on humans as they not only affect appearance but have also been linked to physical and mental health. Presently, drugs such as minoxidil and finasteride are used to prevent hair loss or promote hair growth. However, their effect on hair growth wears off upon withdrawal. The use of these drugs is impeded by the trend of the need for natural products extracted from natural sources such as plants. Plant extracts contain a variety of compounds, such as terpenoids, flavonoids, polyphenolic molecules, carotenoids, and fatty acids, which are known to have positive effects on skin cells (Działo et al., 2016). Unsurprisingly, plant extracts have been used to prevent hair loss and promote hair growth since ancient times. Plant extracts are less likely to be toxic and are easy to obtain at a low cost.

A variety of plant extracts have been confirmed to promote hair growth and prevent hair loss. Although the specific mechanisms of some of these extracts remain elusive, current studies have shown that they are non-toxic, have little to no side effects, and can be acquired and purified easily. To date, nearly a thousand plant extracts have been studied in promoting hair growth, and some show significant effects on hair growth (Choi, Boo & Boo, 2024). Some herbal compounds have been incorporated into hair tonics, hair growth promoters, conditioners, shampoos and other hair products to treat hair loss, dry scalp, and lice infection (Roy, Thakur & Dixit, 2008). These compounds mediate their effects by regulating growth factors in the skin. Roh et al. (2002) showed that *Sophora flavescens* extract promotes hair growth by inducing and regulating the expression levels of IGF-1 and KGF in dermal cells. Additionally, the extracts also inhibited type II 5α-reductase activity, an effect which was also essential for stimulating hair growth (Roh et al., 2002). Peppermint (*Mentha piperita*) is another commonly used plant extract in cosmetic and hair products. Investigations by Oh, Park & Kim (2014) demonstrated that 3 percent peppermint oil is a more effective stimulator of hair growth compared to minoxidil of the same concentration. Peppermint oil increases the expression of alkaline phosphatase (ALP) and IGF-1, which in turn induces hair growth (Oh, Park & Kim, 2014). Linololinic acid (LA) extracted from *Malva verticillate* seeds has also been shown to upregulate KGF, VEGF, HGF, and IGF-1 and promote hair growth in a dose-dependent manner (Ryu et al., 2021). Ryu et al. (2021) showed that LA induces the expression of cell cycle-related proteins such as cyclin D1 and cyclin-dependent kinase 2, and upregulates the Wnt/β-catenin protein signaling pathway, thus, inducing the growth of human follicles dermal papilla cells (HFDPCs) and hair growth. *Tectona grandis Linn.* seed extracts are used as a hair tonic in Indian traditional medicine and have been demonstrated to induce hair growth similarly to minoxidil (Jaybhaye et al., 2010). Red ginseng has a variety of effects such as antioxidant, anti-tumor, anti-mutagenic, and anti-diabetes; the main active ingredient is ginsenoside. Park et al. (2015) found that red ginseng extract and ginsenoside can enhance the proliferation of human dermal papilla cells (hDPCs), activate ERK and AKT signaling pathways in hDPCs, and up-regulate the proliferation of hair matrix keratinocytes, and also inhibits dihydrotestosterone (DHT) -induced androgen receptor transcription, thereby promoting human hair growth. Extracts from *Geranium sibiricum* are used to treat diarrhea, bacterial infection, and cancer among other conditions. Boisvert et al. investigated the effects of *geranium sibiricum* extract on hair growth and found that *geranium sibiricum* extract significantly reduces the number of mast cells and the expression of transforming growth factor-β(TGF-β) in mouse skin while promoting the expression of HGF and VEGF (Boisvert et al., 2017). By regulating the expression of growth factors and cellular responses, *geranium sibiricum* extract promotes hair growth (Boisvert et al., 2017). Other plant extracts that have been shown to promote hair growth include extracts from *Miscanthus sinensis var. purpurascens* (MSP) and *Carthamus tinctorius*. Jeong et al. (2018) showed that methanol extract from MSP upregulates the expression of HGF and β-catenin in hDPCs and downregulates the expression of TGF-β. Additionally, the extract facilitates hair follicle transition from the resting phase to the growth phase (Jeong et al., 2018). Ethanol extract of *Carthamus tinctorius* induces dermal and HaCaT cell proliferation and significantly stimulates the expression of hair growth-promoting factors, such as VEGF and KGF. Additionally, the ethanol extract inhibited the expression of TGF-β while significantly increasing the length of cultured hair follicles (Junlatat & Sripanidkulchai, 2014). Extracts from *Salvia plebeia*, a traditional herbal medicine used to treat hepatitis, menorrhagia, diarrhea, hemorrhoids and other diseases, have also been demonstrated to promote hair growth. Jin et al. (2020) found that the extract of *Salvia plebeia* can activate proliferation in human dermal cells and promote hair growth by increasing HGF expression and inhibiting TGF-β1 and SMAD2/3 signaling. Shikimic acid (SA), which is present in plant stem cells, has also been demonstrated to promote hair growth and elongation in mouse models by initiating the proliferation of human dermal papilla cells (hDPC) and human outer root sheath cells (hORSC) (Choi et al., 2019). This has led to it being suggested as a potential treatment for alopecia (Choi et al., 2019). In addition to shikimic acid, sinapic acid, caffeine, proanthocyanidins B-3, and ginsenoside Rb1, have all been shown to promote hair growth and elongation (Park & Lee, 2021). Substances such as shikimic acid and sinapic acid contained in plants can regulate hair follicle development and promote hair growth by regulating signaling pathways related to hair follicle development (Choi et al., 2019; Woo et al., 2017). KGF-2 can significantly promote hair follicle development and hair growth. Using plant expression systems to express KGF-2 can explore the synergistic therapeutic effect of plant-derived KGF-2 and active ingredients in plants.

Although the specific mechanism of action of some plant extracts has not been studied, they are non-toxic and have few side effects, are easy to obtain, in line with the current pursuit of pure natural cosmetics and effective products, and have multiple advantages. Taken together, plants already contain multiple active molecules that can promote hair growth, of which some are used in products already on the market. Coupling the expression of recombinant proteins known to promote hair growth in these plants can lead to better hair growth products.

### Feasibility of synthesizing TD1-KGF-2 using molecular agriculture

Currently, the expression of recombinant proteins is mainly done in prokaryotic systems or yeast, which has several drawbacks propagated by endotoxin, pathogen contamination, and cost of production. The expression of pharmacologically relevant proteins in plant bioreactors, promises the evasion of these drawbacks while reducing the cost of production. The use of plant bioreactors is also advantageous, as many plant extracts contain useful compounds that promote skin health and hair growth. Fusion protein expression in the plant system can play a synergistic effect of the active components in the plant and foreign proteins, so as to obtain a better therapeutic effect.

A potential model plant for expressing fusion KGF-2 is *A. thaliana*. Compared with other plant systems, *A. thaliana* has a clear genetic background and abundant research resources, which is suitable for studying the expression mechanism of foreign proteins. *A. thaliana* is a model plant with many advantages due to its fast growth rate, short growth cycle, strong adaptability, high genetic transformation efficiency, and large whole-plant biomass (Zheng et al., 2021). *A. thaliana* extracts are used in cosmetic products, as they contain flavonoid glycoside derivatives, monophenolic compounds, polyphenolic compounds, phenolic acids, and alkaloids among other substances. Additionally, *A. thaliana* contains substances such as anthocyanins, shikimic acid, and quercitrin, which are known to promote hair growth (Park & Lee, 2021; Mattioli et al., 2019). Thus, *A. thaliana* is an ideal bioreactor for KGF-2 fusion protein expression, as its content can have synergistic effects with KGF-2, making the extract a better therapeutic. At present, TD1 has a clear transdermal mechanism and related research basis for transdermal efficiency. The fusion expression of TD1 and KGF-2 can enhance the transdermal ability of KGF-2.

**Figure 1:**
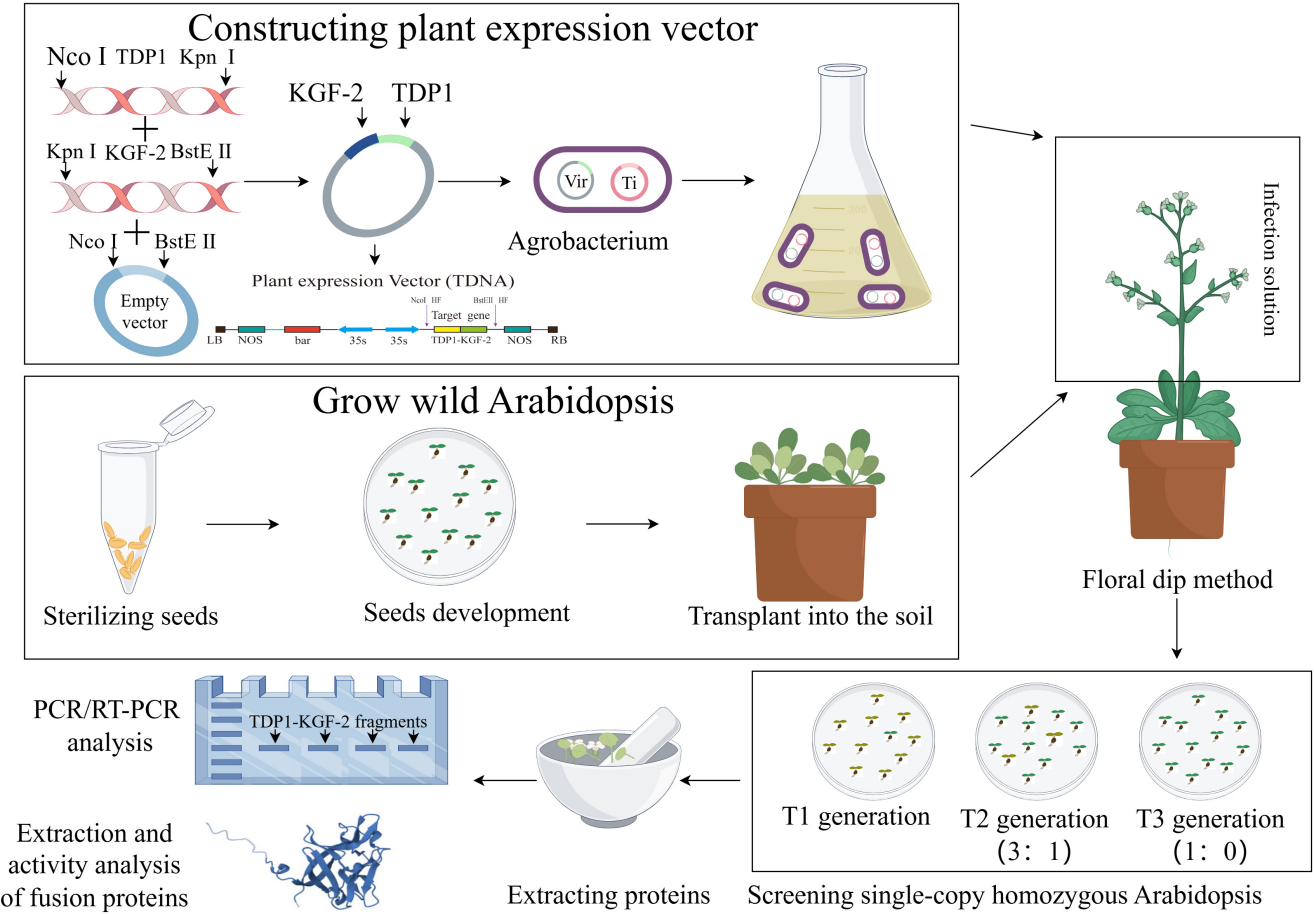
Schematic representation of *A. thaliana* expressing TDP1-KGF-2 by figdraw.com.

In order to ensure the stable expression of TD1 in plants, we will optimize the preference of the TD1 codon and change part of its amino acid residues through point mutation to obtain the TD1 mutant transdermal peptide TDP1. TDP1 is fused to the KGF-2 gene that is also subjected to codon optimization. In the preliminary design, a flexible linker is inserted between the two protein molecules to prevent the structure of the two protein molecules from affecting the function of the fusion protein (Chen, Zaro & Shen, 2013). Selecting appropriate plant expression vectors that contain an enhanced 35S promoter and 35S terminator will increase the accumulation of the fusion protein TDP1-KGF-2. To enhance the transdermal ability of KGF-2 and overcome the difficulties of downstream purification, we selected two fusion protein tags, His-tag and TDP1. Inflorescence transformation can integrate the target gene into the plant genome and stabilize inheritance and whole-plant expression. The *A. thaliana* inflorescence was infected with Agrobacterium tumeticum and the progeny were screened until the single copy homozygous transgenic *A. thaliana* was obtained in the third filial generation. Figure 1 is a schematic representation of *A. thaliana* expressing TDP1-KGF-2. In future studies, the feasibility of TDP1-KGF-2 expression in a whole plant system will be tested in *A. thaliana*. The expression of fusion protein TDP1-KGF-2 in *A. thaliana* plant expression system does not cause endotoxin and other problems, and the fusion protein TDP1-KGF-2 can be obtained at a lower cost by optimizing planting conditions and other operations. This enhances the transdermal delivery of KGF-2 while further reducing production costs, thereby increasing its commercial value and applications. The transdermal ability and biological activity of TDP1-KGF-2 will also be evaluated, and hopefully, this will provide a new application model of plant bioreactor in the field of hair growth products for exploring new ideas for the development of natural hair growth raw materials. Plant bioreactors have a broad application prospect in this field.

## Outlook

Molecular farming has been investigated for more than 40 years and since then has been used to produce vaccines and antibodies. This the past decade, molecular farming has been explored for the expression of cytokines and growth factors. Now, the advantages and disadvantages of plant bioreactors have been clearly revealed, and it is most appropriate to use plants to express proteins that meet the concept of pure nature and with no purity requirements. Although there are some problems in the expression of exogenous proteins in plant expression systems, such as low expression amount, post-translational modification, transformation efficiency, horizontal gene transfer, and ecological impact, these problems can be solved by optimizing codon bias, co-expression of molecular chaperones, optimizing post-translational modification, optimizing transformation methods, and precise gene editing. Many plants contain a variety of active ingredients, which can be used in many fields such as the treatment of diseases, skincare, hair care, and so on. The expression of fusion proteins, such as TDP1-KGF-2, in *A. thaliana* and exploration of its biological activity can lead to further understanding of the vast potential of plant bioreactors. Plant-derived TDP1-KGF-2 has advantages such as natural origin, high safety, low cost, and sustainability. It can meet the needs of consumers for hair growth, anti-aging, skin repair and other products, and provide new raw material options for the cosmetics industry. Expression of TDP1-KGF-2 using a plant expression system can significantly improve the practical application value and commercial potential of KGF-2. Additionally, it can provide the relevant basis and support for the use of plants as expression systems, especially plants that are known to contain active compounds with beneficial effects on hair growth and health. The expression of TDP1-KGF-2 in *A. thaliana* undoubtedly leads to synergistic effects conferred by the fusion protein and active compounds in *A. thaliana*. There is a need to further explore new application directions with either different growth factors or different plants, with a focus on efficacy, cost, and market application. Other potential plant expression systems include *Geranium sibiricum* and *Malva verticillate*, which are not only known to have active ingredients that promote hair health, but also have a high whole-plant biomass and high growth rate. Currently, few studies have been conducted to explore the synergistic effect of KGF-2 or other proteins known to promote hair growth and plant extracts. Although there is now more pursuit of pure natural concepts in the market for hair growth, hair care, hair loss prevention and other products. Aside from the expression of hair growth-related proteins, plant bioreactors can be used to express proteins known to treat other diseases. For plants that have been confirmed to contain effective ingredients for treating certain diseases, such as *Sophora flavescens*, which contains effective ingredients for promoting hair growth, and *calendula officinalis*, which contains effective ingredients for anti-tumor treatment (Jiménez-Medina et al., 2006), can be used as expression systems for molecules that treat these diseases. The appropriate plant expression system can be selected by considering multiple factors such as target protein, plant characteristics, and active ingredients contained in plants. After exogenous genes are introduced into plants, plants with high expression can be obtained by optimizing post-translational modification and other operations. At the same time, regulatory constraints and the reduction of production costs by optimizing cultivation conditions and extraction processes should be considered to further enhance their market feasibility. This will enable the synthesis of synergistic therapeutics with enhanced treatment efficacy. The use of plants as bioreactors has immense and wide application potential.

## Additional Information and Declarations

### Data Availability

The following information was supplied regarding data availability: This is a literature review.
